# Performance on the Robotics On-Board Trainer (ROBoT-r) Spaceflight Simulation During Acute Sleep Deprivation

**DOI:** 10.3389/fnins.2020.00697

**Published:** 2020-07-21

**Authors:** Lily Wong, Sean Pradhan, John Karasinski, Cindy Hu, Gary Strangman, Vladimir Ivkovic, Lucia Arsintescu, Erin Flynn-Evans

**Affiliations:** ^1^Fatigue Countermeasures Laboratory, Human Systems Integration Division, San José State University Research Foundation, Moffett Field, CA, United States; ^2^Department of Management, Menlo College, Atherton, CA, United States; ^3^Department of Mechanical and Aerospace Engineering, University of California, Davis, Davis, CA, United States; ^4^Human Systems Integration Division, NASA Ames Research Center, Moffett Field, CA, United States; ^5^Department of Psychiatry, Massachusetts General Hospital, Charlestown, MA, United States; ^6^Department of Psychiatry, Harvard Medical School, Boston, MA, United States; ^7^Fatigue Countermeasures Laboratory, Human Systems Integration Division, NASA Ames Research Center, Moffett Field, CA, United States

**Keywords:** Robotics On-Board Trainer, performance, complex tasks, sleep loss, circadian phase

## Abstract

Exploration of deep space poses many challenges. Mission support personnel will not be immediately available to assist crewmembers performing complex operations on future long-duration exploration operations. Consequently, it is imperative that crewmembers have objective, reliable, and non-invasive metrics available to aid them in determining their fitness for duty prior to engaging in potentially dangerous tasks. The Robotics On-Board Trainer (ROBoT) task is NASA’s platform for training astronauts to perform docking and grappling maneuvers. It is regularly used by crewmembers during spaceflight for refresher training. The operational ROBoT system, however, does not record data. Thus, a research version of ROBoT, called ROBoT-r, was developed so that operationally relevant data could be mined to provide feedback to crewmembers. We investigated whether ROBoT-r metrics would change according to sleep loss and circadian phase in a 28-h laboratory-based sleep deprivation study. Overall, participants showed improvement over time despite sleep loss, indicating continued learning. Performance on the psychomotor vigilance task (PVT) followed an expected profile, with reduced performance across the night. These findings suggest that individuals may be able to temporarily compensate for sleep loss to maintain performance on complex, novel tasks. It is possible that some ROBoT-r metrics may be sensitive to sleep loss after longer bouts of wakefulness or after individuals have habituated to the task. Studies with additional participants and extended pre-training on the ROBoT-r task should be conducted to disentangle how brain activity may change as individuals learn and habituate to complex tasks during sleep loss.

## Introduction

Alertness and performance fluctuate according to prior sleep history and circadian time of day (Dijk et al., 1992). Crewmembers aboard the International Space Station (ISS) and Space Shuttle sleep less in space relative to on Earth (Barger et al., 2014; Gonfalone, 2016) and they spend approximately 1 out of every 5 days in a circadian misaligned state (Flynn-Evans et al., 2015). In addition, they report sleeping less on nights prior to critical tasks and can be required to “slam shift,” staying awake for many hours to adjust their sleep schedules in advance of a rendezvous with a visiting vehicle (Barger et al., 2014). The degree of sleep loss and circadian misalignment experienced by astronauts is unclear, but similar patterns of long bouts of waking and short sleep episodes have been associated with attentional failures and performance impairment among other high-performing cohorts on Earth (Lockley et al., 2004). Such degradation has been equated to performance impairment due to alcohol intoxication (Dawson and Reid, 1997). There is some evidence to suggest that sleep loss and circadian misalignment have impacted operations during spaceflight. In particular, the catastrophic collision of the Progress resupply vehicle with the Space Station Mir was attributed in part to human error due to a crewmember experiencing sleep loss and fragmentation prior to the accident (Arthur, 1998).

Exploration of deep space poses additional complications in managing crewmember fatigue. Mission support personnel will not be immediately available to back up and correct crewmembers performing complex operations due to the expected communication delay and lack of situational awareness. Similarly, mission support will not be immediately available to assist in decision-making following an incident or accident. As a result, it is imperative that crewmembers have objective and reliable metrics available to assist them in identifying performance risks prior to engaging in potentially dangerous mission operations.

Although there are several cognitive tests that have been identified as being associated with fatigue (e.g., the psychomotor vigilance task; PVT; Dinges and Powell, 1985), such tests have been questioned for their operational relevance. An ideal performance indicator for spaceflight should be sensitive to sleep loss and circadian misalignment using measures that are similar to the crewmember’s actual work requirements. The Robotics On-Board Trainer (ROBoT), derivative of NASA’s Dynamic Skills Trainer (DST; Johnson and Alexander, 2013) is just such an operational measure of an individual’s performance capabilities. ROBoT simulates a track-and-capture maneuver using Canadarm2 robotic arm on the ISS to grapple a transiting spacecraft. The research version of ROBoT, ROBoT-r, was recently developed and has been used in NASA analog missions (Ivkovic et al., 2019), but changes in performance using ROBoT-r under controlled conditions of sleep loss and circadian misalignment have not been characterized. Thus, the goal of this investigation was to identify changes in performance on ROBoT-r over 28 h of wakefulness. Specifically, we aimed to test the hypothesis that three measures of performance extracted from ROBoT-r (% of successful captures, alignment-reversal score, and efficiency to capture, see methods for detailed descriptions of these measures) would decline over 28 h of sleep loss. Furthermore, we aimed to compare ROBoT-r outcomes with the PVT, which is currently a “gold-standard” measure of performance impairment arising from fatigue.

## Materials and Methods

### ROBoT-r Simulation

**FIGURE 1:**
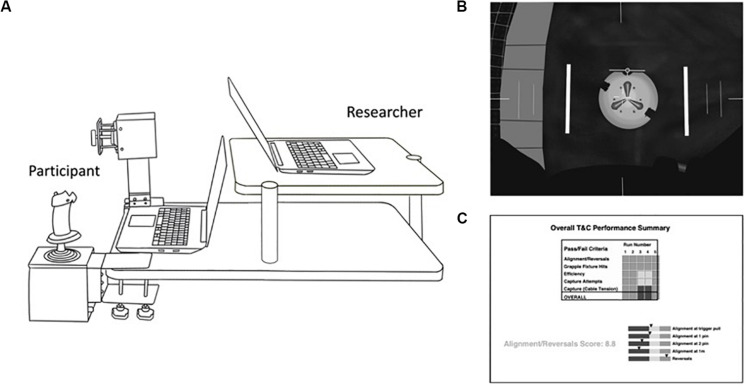
Experimental Set-up and screen views. **(A)** Illustration of the ROBoT-r experimental set-up. The participant sat on the left side and used the rotational (near side, used by participant’s right hand) and translational (far side, used by participant’s left hand) hand controllers to move the robotic arm toward the target. **(B)** The view of the screen facing the participant. The white circle in the center represents the target that the participant must grapple. In order to achieve the grapple, the participant must align the end of the robotic arm, represented by the thin horizontal line just above the target, with the center of the target. **(C)** The view of the screen facing the investigator showing the individual’s performance, including whether the individual captured the target, the alignment/reversal score, and efficiency to capture.

We assessed performance on ROBoT-r in the Fatigue Countermeasures Laboratory at NASA Ames Research Center (NASA-ARC). ROBoT-r is used by astronauts to rehearse docking and grappling maneuvers using the robotic arm on the ISS. The simulation is based on highly realistic 3D simulations of the Canadarm2 robotic arm on the ISS and associated physics relating to spaceflight (Figure 1). The ROBoT-r simulation involves a difficult and critical spaceflight maneuver of grappling an incoming spacecraft. To complete the task, the participant must extend the robotic arm to the incoming spacecraft, line up the end effector with a target on the approaching vessel, and grapple a pin on the vessel to “capture” the target. This maneuver requires situation analysis, planning, decision-making, object orientation, mental rotation, visual processing, fine motor control, and visual motor integration.

Each ROBoT-r testing session consists of 12 runs that range from easy to hard H-II Transfer Vehicle (i.e., resupply vessel) track-and-capture scenarios. The simulation randomly selects a run from a large library in each category prior to the presentation of the visual display. Participants have up to 99 s to successfully grapple the target before the run times-out. Trials can be administered with “frozen” or “unfrozen” dynamics. That is, the task can be completed with the spacecraft target grapple fixture locked into a fixed position in space (frozen dynamics) or the task can be completed with the spacecraft target (and connected grapple fixture) moving independently, as would be the case during a real track-and-capture maneuver (unfrozen dynamics). Participants completed all experimental trials using unfrozen dynamics.

The physical ROBoT-r system includes a left-hand translational controller (x/y/z directions) and a right-hand rotational controller (pitch/roll/yaw), plus two laptop computer screens. One screen displays a realistic rendering of the entire 3D scene, including docking/grappling target and arm, from three separate viewpoints. A second screen displays graphs and status indicators for monitoring the arm status and user’s performance in real-time. During our experiment, the second screen was oriented toward the study staff member in order to monitor the participant’s progress. In spaceflight operations, crewmembers would be able to view the second screen. Figure 1 shows the orientation of the hand controllers and screens. The current study was approved by the NASA Ames Human Research Institutional Review Board (protocol HRI-339).

### Study Information

#### Participants and Selection/Exclusion Criteria

Participants were required to be healthy (i.e., certified to participate in the study by their primary care physician, and cleared by the NASA Ames Medical Monitor following medical record review), drug and medication free, non-smokers, and have no history of serious chronic conditions or mental illness. To participate, individuals were expected to have normal sleep habits defined as Pittsburg Sleep Quality Index (PSQI; Buysse et al., 1989) scores less than five, Fatigue Severity Scale (Krupp et al., 1989) scores less than 36, and Morningness-Eveningness Questionnaire (MEQ; Horne and Östberg, 1976) scores less than 58 or greater than 42. Participants were excluded from participation if they had experienced acute total sleep deprivation (one night of staying awake all night) anytime in the prior 12 months, or if they traveled across one or more time zones in the prior 3 months.

Participants were also excluded if they typically consumed excessive alcohol (i.e., greater than 14 standard drinks/week for males, and greater than 7 standard drinks/week for females), and if they reported illicit drug use. Similarly, participants were excluded if they scored higher than 70 on the Minnesota Multiphasic Personality Inventory (MMPI-2; Butcher et al., 1989). Depression scale, higher than 75 on the MMPI-2 Psychopathic Deviance, Schizophrenia, and Hypomania State scale, higher than 10 on the Beck Depression Inventory (BDI; Beck et al., 1961), or higher than 40 on the State Trait Anxiety Inventory (Spielberger et al., 1983). Additionally, participants were excluded if they scored above any of the following criteria on the scales of the Symptom Checklist 90-R (Derogatis and Savitz, 1999): greater than 1.25 on Depression, greater than 1 on Hostility, greater than 0.75 on Phobic Anxiety, greater than 1.25 on Paranoid Ideation, greater than 1 on Psychoticism, and greater than 1.25 on Anxiety.

### Pre-study Procedures

One week before the laboratory study, participants maintained stable and individually selected sleep-wake schedules, with 8 h in bed each night. To ensure compliance, each participant wore an activity monitor (Actiwatch Spectrum, Respironics Inc^®^, Bend, OR, United States) on their non-dominant wrist, recorded daily time stamped voicemails at their sleep and wake times, and maintained a sleep diary. Each participant completed five 1–2 h training sessions on the ROBoT-r simulator on five separate days during the week prior to the laboratory visit. During the first training session, prior to attempting their first run, participants were oriented to the simulator and provided with basic information on how the robotic arm moves through space (e.g., definitions of pitch, yaw, roll, and relationship to the hand controllers). Each training session included 12 ROBoT-r runs, with three easy runs, three medium-easy runs, three medium-hard runs, and three hard runs, defined as 25, 50, 75, and 100% of the maximum linear velocity and angular velocity allowed by NASA for capture. The first training session was completed with frozen dynamics (i.e., a stationary target) in order to orient the participant to the simulation. Subsequent training sessions were completed with unfrozen dynamics (i.e., the capsule constituted a moving target).

#### Laboratory Procedures

Participants arrived at the NASA-ARC sleep laboratory 1–2 h after their habitual wake time. Following orientation, participants were placed in a time-free environment and instructed to select a comfortable chair on wheels to sit in, where they maintained a constant posture throughout the study. In order to maintain a regulated physiological state, participants were prohibited from standing during the study. In order to complete study procedures, participants were wheeled from a common room to a sound-attenuated room that housed the ROBoT-r simulator during trials. Similarly, participants were wheeled to the bathroom as needed and instructed to use the minimal posture change required to move to the toilet for bathroom breaks. In order to reduce the influence of metabolism on alertness, meals were divided into hourly isocaloric snacks based on the participant’s body weight. Ambient light was maintained at a constant illuminance of less than 15 lux, with an irradiance level measured at 1.03 μW/cm^2^ at the angle of gaze, and all extraneous lighting sources were prohibited. Study participants were run in groups of up to four participants at a time.

The ROBoT-r simulation was administered regularly, with each participant completing a 12-run session approximately every 3–4 h over 28 h. A trained study staff member administered the sessions and provided verbal feedback to the participant following each run. Feedback consisted of informing the participant about the position of the grapple fixture over the pin (e.g., too far over, too shallow, the angle of the grapple over the pin), time to capture, alignment to the pin, number of reversals and alignment-reversal score. The Karolinska Sleepiness Scale (KSS; Åkerstedt and Gillberg, 1990) and a five-minute version of the PVT (administered on a PVT-192 device) were completed in a separate, quiet room immediately following each ROBoT-r session.

### Statistical Analyses

Analyses were calculated using R statistical software (version 3.3.3, R Core Team, 2014) and SPSS (version 26.0, IBM SPSS Statistics for Windows, Armonk, NY, United States). We calculated the following PVT metrics: (1) mean (1/RT)*1000 (reciprocal reaction time, or response speed), (2) *number of lapses* – the cumulative number of reaction times exceeding 500 ms, (3) *optimum response times* – the fastest 10% of response times for all trials (fastest 10% mean RT) which indicates the best performance a participant is capable of producing, and (4) *cognitive slowing* – the slowest 10% of reciprocal response times for all trials (slowest 10% mean (1/RT)*1000), which indicates the vigilance response slowing. A PVT response was considered valid if RT was greater than 100 ms. Responses with an RT less than 100 ms were counted as false starts. All PVT data were analyzed using linear mixed-effects models including participant as a random effect. We further computed conditional and marginal pseudo *R*^2^ in order to provide a measure of the effect size.

We evaluated three primary measures from the ROBoT-r trials including the alignment-reversal score, the efficiency to capture, and the percentage of successful captures. The alignment-reversal score is a composite score ranging from 0 to 10 (10 is best) that is automatically calculated by the ROBoT-r software following each trial based on how the end effector is aligned with the target at pre-defined distances from the target. Efficiency to capture is the duration of time measured in seconds that it takes for an individual to complete the task (or 99 s to time out if they were not successful) and “successful capture” is simply whether or not the participant was able to capture the target within the allotted time, irrespective of the quality of the capture. We evaluated each of these measures over all runs (i.e., the mean of an entire trial including all difficulty levels), by time in the 28-h study, and by difficulty level (i.e., easy, medium-easy, medium-hard, hard).

We compared each of the ROBoT-r outcomes to the PVT outcomes and self-reported sleepiness using Pearson correlations. We evaluated trends over time for the pre-laboratory training sessions and for the laboratory stay separately for ROBoT-r and PVT outcomes, using mixed-effects linear regressions (with participant as the repeated-factor). To further investigate participants’ learning on the ROBoT-r task, we performed a series of polynomial mixed-effects linear regressions using the following primary metrics as outcomes: *efficiency* (i.e., time in seconds), *alignment-reversal scores*, *captures*, and *overall success*. In order to parse out the impact of learning effects, we controlled for each individual subjects’ variance by including subject as a random effect term across the models. Given the binary nature of captures and overall success, a binomial family parameter was specified for all models containing these variables. Mixed-effects models for the ROBoT-r outcomes were performed in RStudio (Version 1.1.456, Boston, MA, United States) for macOS using the lme4 (Bates et al., 2015) and lmerTest packages (Kuznetsova et al., 2017). Both conditional and marginal pseudo *R*^2^ values were computed for these models using the MuMIn package (Bartoń, 2009). Conditional pseudo-*R*^2^ describes the proportion of variance explained by both the fixed and random effects, while marginal pseudo-*R*^2^ indicates variance explained by the fixed factors alone (Nakagawa and Schielzeth, 2013).

## Results

Nine participants completed the study (4 female, 5 male; *M*_*age*_ = 31.89 years, SD = 10.04, Range = 19–49). The demographic characteristics of the participants are shown in Table 1.

**TABLE 1:** Demographic information.

Variable	*M* (SD)	Range
Sleep duration (h)	8.08 (0.45)	7.35–9.52
Pre-study bedtime	22:36 (0:52)	21:04–01:57
Pre-study waketime	06:41 (1:00)	05:06–10:34
PSQI	2.78 (1.64)	0.00–5.00
MEQ	53.39 (6.16)	43.00–61.00

*Sleep duration was calculated using the sleep diary. *M*, mean; SD, standard deviation; h, hours; PSQI, Pittsburgh Sleep Quality Index; MEQ, Morningness-Eveningness Questionnaire.*

### ROBoT-r Performance

**FIGURE 2:**
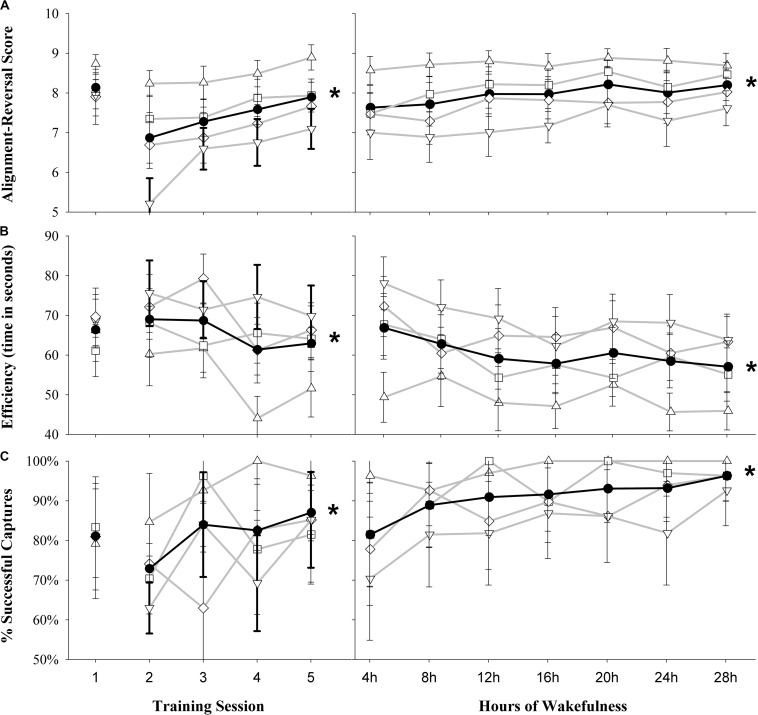
Overall performance on ROBoT-r for the group for the five training sessions (left) and by time awake during the study (right). **(A)** Alignment-reversal score, **(B)** efficiency (seconds to capture), **(C)** percentage of successful captures. Filled black circles and black line indicates the overall mean of all trials. Sub-sets of trials by difficulty are joined by gray lines; open triangles, easy trials; open squares, medium-easy trials; open diamonds, medium hard trials; open inverted triangles, hard trials. Note that training session 1 was completed with frozen dynamics (i.e., without motion) and is excluded from analyses. Plots represent mean ± standard error, *p < 0.05 for the overall change in time from the linear mixed effects models.

The participants showed a significant improvement from the second to the last training session (the first training session was excluded due to it being completed with frozen dynamics) for all measures indicating a practice effect (alignment-reversal score *b* = 0.35, SE = 0.07, *p* < 0.001, Conditional Pseudo-*R*^2^ = 0.10, Marginal Pseudo-*R*^2^ = 0.05; efficiency to capture *b* = −2.60, SE = 0.91, *p* = 0.004, Conditional Pseudo-*R*^2^ = 0.25, Marginal Pseudo-*R*^2^ = 0.01; percentage of successful captures *b* = 0.30, SE = 0.12, *p* = 0.01, Conditional Pseudo-*R*^2^ = 0.13, Marginal Pseudo-*R*^2^ = 0.03; Figure 2, left panels). During the study, there was also a modest, but statistically significant improvement in performance over time for the overall alignment-reversal score (*b* = 0.10, SE = 0.02, *p* < 0.001, Conditional Pseudo-*R*^2^ = 0.15, Marginal Pseudo-*R*^2^ = 0.01; see Figure 2A, right), efficiency to capture (*b* = −1.16, SE = 0.34, *p* = 0.001, Conditional Pseudo-*R*^2^ = 0.25, Marginal Pseudo-*R*^2^ = 0.01; see Figure 2B, right), and percentage of successful captures (*b* = 0.25, SE = 0.06, *p* < 0.001, Conditional Pseudo-*R*^2^ = 0.17, Marginal Pseudo-*R*^2^ = 0.06; see Figure 2C, right) according to our mixed-effects regression analysis. In order to determine whether differences might be apparent depending on the difficulty of the task, we separated the captures by difficulty level and found several differences in the ROBoT-r metrics by time awake (Figure 2, gray lines and Table 2). Specifically, we found a modest, but statistically significant improvement in the percentage of successful captures for medium-easy and hard trials. We also found a significant improvement in the alignment reversal scores and efficiency to capture (i.e., decreased time) for medium-easy and hard trials.

**TABLE 2:** Mixed-effects regression models for ROBoT-r outcomes over time awake by difficulty level.

	Trial difficulty															
	Easy				Medium-easy				Medium-hard				Hard			
Outcome	*b* (SE)	*p*	$R_{C}^{2}$	$R_{M}^{2}$	*b* (SE)	*p*	$R_{C}^{2}$	$R_{M}^{2}$	*b* (SE)	*p*	$R_{C}^{2}$	$R_{M}^{2}$	*b* (SE)	*p*	$R_{C}^{2}$	$R_{M}^{2}$
Alignment-reversal score	0.03 (0.03)	0.38	0.17	0.003	0.13 (0.04)	0.001	0.21	0.04	0.09 (0.05)	0.06	0.17	0.01	0.13 (0.06)	0.02	0.18	0.02
Efficiency to capture	−0.68 (0.51)	0.19	0.18	0.02	−1.43 (0.61)	0.02	0.30	0.02	−0.78 (0.67)	0.24	0.24	0.005	−1.76 (0.64)	0.007	0.31	0.02
Percentage of successful captures	0.76 (0.40)	0.06	0.31	0.02	0.39 (0.16)	0.02	0.31	0.02	0.22 (0.11)	0.06	0.08	0.05	0.20 (0.10)	0.05	0.20	0.04

*$R_{C}^{2}$, Conditional Pseudo-*R*^2^; $R_{M}^{2}$, Marginal Pseudo-*R*^2^.*

### PVT Analyses

**FIGURE 3:**
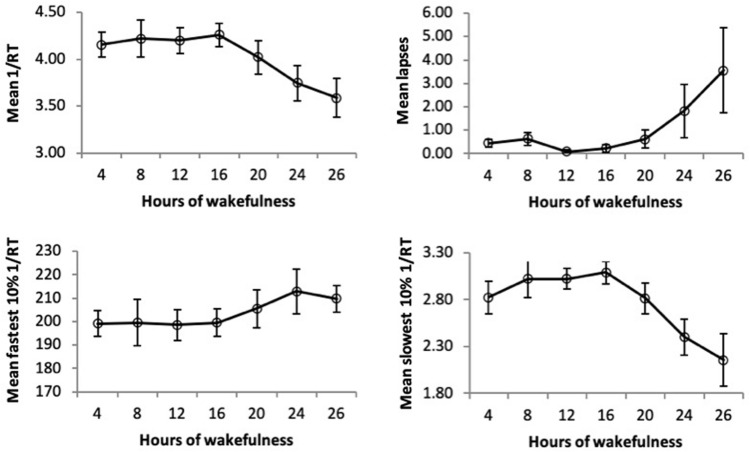
Change in PVT outcomes by time awake. Open symbols represent mean ± standard error. 1/RT = reciprocal reaction time, lapses = RT > 500 ms.

Performance on the PVT varied as expected, with worsening performance over time awake (Figure 3). For mean 1/RT there was a significant decrease over time (*b* = −0.13, SE = 0.02, *p* < 0.001, Conditional Pseudo-*R*^2^ = 0.84, Marginal Pseudo-*R*^2^ = 0.03). Analysis of lapses indicated an increase of lapses over time (*b* = 1.30, SE = 0.30, *p* < 0.001, Conditional Pseudo-*R*^2^ = 0.76, Marginal Pseudo-*R*^2^ = 0.15). The analyses also revealed an increase in fastest 10% RT (*b* = 3.26, SE = 0.67, *p* < 0.001, Conditional Pseudo-*R*^2^ = 0.88, Marginal Pseudo-*R*^2^ = 0.11) and a decrease in slowest 10% 1/RT over time (*b* = −0.18, SE = 0.03, *p* < 0.001, Conditional Pseudo-*R*^2^ = 0.56, Marginal Pseudo-*R*^2^ = 0.09). However, with the exception of a positive relationship between sleepiness ratings and the percentage of successful captures (*r* = 0.24, *p* < 0.05), we found no other significant associations between any of the PVT outcomes or KSS ratings and ROBoT-r performance metrics (*r* < ± 0.21, *p* > 0.05; non-significant results shown in Supplementary Table S1).

### Learning Effects

None of the models showed a significant effect when controlling for learning effects (*p* > 0.05), except for the effect of time on the efficiency for medium-easy trials (*p* = 0.03, Conditional Pseudo-*R*^2^ = 0.24, Marginal Pseudo-*R*^2^ = 0.01, i.e., performance slowed during the night; full results shown in Supplementary Table S2).

## Discussion

We evaluated performance on the ROBoT-r during a laboratory sleep deprivation study. Although performance on the PVT changed as expected, with worsening performance over time awake, ROBoT-r performance outcomes did not follow a similar pattern. We observed a modest improvement in performance on ROBoT-r over the course of sleep loss. We did not observe any correlations between ROBoT-r performance and PVT outcomes for our study. These findings were contrary to expectations. We hypothesized that performance on ROBoT-r would worsen, with fewer successful captures, and that the time to complete the maneuver would be longer over the course of sleep loss relative to baseline rested performance. We instead found that alignment-reversal score performance, capture success, and time to complete the trials modestly improved over time. Our findings support the notion that individuals who are acutely sleep deprived can maintain performance on this interesting, complex task.

Despite the unexpected results, our findings are similar to prior studies. Strangman et al. (2005) evaluated a 3D sensorimotor docking task following normal sleep and 24 h of sleep deprivation. During this study, six individuals completed a simulated docking maneuver, which required them to fly a remote control spacecraft into the center of a computer screen using joysticks, while in a Magnetic Resonance Imaging (MRI) scanner. The investigators found no performance reduction on any of the metrics evaluated. However, they did find differences in brain activity during sleep deprivation, suggesting that individuals experienced an adaptive cerebral response when attempting to perform a complex task while sleep deprived. Studies that have been conducted to evaluate physical performance on robotic laparoscopic procedures during sleep deprivation have yielded similar results (Howard et al., 2003; Uchal et al., 2005; Lehmann et al., 2010; Neuschwander et al., 2017). The motor coordination and mental focus required to complete laparoscopic surgery procedures is similar to that required to complete a robotic arm maneuver. During laparoscopic surgery, the surgeon must use two hands and a camera display to manipulate robotic instruments inserted into a patient to complete the procedure. In a protocol similar to that used in the present study, with five pre-study training sessions on a laparoscopic simulator, one group found no differences in performance in a rested condition compared to a sleep-deprived condition (Lehmann et al., 2010). These findings have been supported by several other studies, with varying study designs (Howard et al., 2003; Uchal et al., 2005; Neuschwander et al., 2017). Although these findings suggest that motivated individuals are able to compensate for sleep loss to complete complex tasks, it appears that such compensation comes at the expense of cognitive reserve in other areas. For example, sleep-deprived surgeons have been shown to exhibit impaired teamwork skills, such as reduced verbal communication with team members, during laparoscopic surgery (Neuschwander et al., 2017).

The notion that individuals can invoke the resources needed to complete complex tasks is also supported by controlled laboratory studies and brain imaging experiments. In a review of the effects of sleep deprivation on decision-making, Harrison and Horne (2000) summarized work suggesting that tasks that are “complex, interesting, or variable…encourage sleepy people to apply compensatory effort and perform normally.” Several studies have been conducted where individuals were sleep deprived and then required to complete complex tasks while simultaneously being scanned using MRI. These studies have demonstrated that individuals are able to compensate for increased task difficulty (Drummond et al., 2000; Chee and Choo, 2004; Drummond et al., 2004) and cognitive load (Choo et al., 2005) during sleep deprivation by recruiting resources from brain regions that are inactive during simple or boring tasks.

It is possible that the novelty of ROBoT-r may wear off after individuals reach an asymptote, which might make some elements of the task vulnerable to fatigue-related reductions in performance. Alternatively, it is possible that once performance plateaus, individuals will exhibit smaller variations in performance, making vulnerability to sleep loss and circadian misalignment easier to detect. We did not have a way to measure either of these possibilities in our study. In addition, we did not conduct enough training simulations for our study participants to reach an asymptote. However, performance on the easy trials appeared to stabilize during the laboratory study. Despite this, we did not see any sleep or time-of-day variation in performance when we restricted our analysis to the easy trials, although the easy trials may not have been challenging enough to elicit mistakes. Additional studies are needed to determine how training effects may have influenced our findings.

It is tempting to deduce that repetitive, standardized reaction time tasks such as the PVT are irrelevant in operational environments where highly trained, motivated individuals are required to complete complex tasks. However, there is evidence that tasks such as the PVT serve as an important—and sensitive—diagnostic tool for identifying vulnerability due to sleep loss. In operational environments, individuals are often required to multi-task and deal with unexpected events. While such variety in workload may be alerting to some extent, the accumulation of multiple layers of vulnerability increases the risk of operational failure (Reason, 2000). For example, high workload, sleep loss, circadian misalignment, and mode confusion are all examples of potential points of vulnerability, but when combined, the risk of operational failure increases. There are numerous examples of accidents that have occurred when highly trained individuals failed to perform while sleep deprived (Mitler et al., 1988; Marcus and Rosekind, 2016). In these cases, it was typically the convergence of many factors that led to the accident, with sleep loss or circadian misalignment being one point of vulnerability. It is unclear, however, what percentage change in response time has operational relevance. Given the challenges with administering a task like the PVT in an operational environment, it may be possible to embed a PVT-like secondary task within the ROBoT-r simulation in order to more passively collect diagnostic information about the state of the operator.

Although we systematically evaluated performance on a complex task over 28 h of sleep deprivation, our study is not without limitation. First, we recruited healthy, astronaut-like individuals to participate in the study. This sample population may be generalizable to other high performing cohorts, such as surgeons, pilots, and drone operators, but the stringent recruiting and small sample of individuals that we studied may not reflect what would occur in the rest of the population. In addition, we only studied one task at four levels of difficulty. There were no circumstances where the participants experienced any off-nominal situations. We also studied performance on the robotic arm simulator in a controlled laboratory environment. The laboratory did not mimic the isolation and confinement or high tempo environment experienced by crewmembers while in space. Future studies should explore whether individuals are still able to maintain alertness in unexpected situations and in operational environments.

## Conclusion

In summary, we attempted to characterize sleep and circadian-related changes in robotics performance. We found that individuals improved on all aspects of performance over time, despite being sleep deprived for over 24 h. We did not find any correlation between ROBoT-r metrics and PVT metrics. Additional studies are needed to determine whether secondary tasks could be embedded within the ROBoT-r task to unmask sleep and circadian-related vulnerabilities. It would also be useful to evaluate how quickly individuals learn ROBoT-r when training sessions are limited to the daytime and under rested conditions, given that the participants were able to continue to improve in their performance during the night. These findings also suggest that it may be valuable to examine ROBoT-r data from individuals who have reached proficiency on the task to determine whether habituation might uncover susceptibility to performance impairment during sleep loss or when performing robotics maneuvers at adverse circadian phases.

## Data Availability Statement

The datasets presented in this article are not readily available because NASA review and ethical approval for secondary use of data are required. Data collected for this study may be made available by the corresponding author after the requestor completes NASA requirements. Requests to access the datasets should be directed to erin.e.flynn-evans@nasa.gov.

## Ethics Statement

The studies involving human participants were reviewed and approved by the NASA Ames Research Center Human Research Institutional Review Board. The patients/participants provided their written informed consent to participate in this study.

## Author Contributions

LW, GS, VI, and EF-E conceptualized the study and analysis. LW, SP, JK, CH, and EF-E collected the data. SP, JK, LA, and EF-E analyzed the data. LW, SP, and EF-E wrote the manuscript. All authors contributed to the article and approved the submitted version.

## Conflict of Interest

The authors declare that the research was conducted in the absence of any commercial or financial relationships that could be construed as a potential conflict of interest.
