# Supplementary material for: Performance on the Robotics On-Board Trainer (ROBoT-r) Spaceflight Simulation During Acute Sleep Deprivation
Source: Front Neurosci. 2020 Jul 21;14:697. doi: 10.3389/fnins.2020.00697 (PMC7385239; doi:10.3389/fnins.2020.00697)
Supplement: Supplementary file 1 [file Table_1.docx]

Supplemental material.

Table 1S. *Correlations between PVT, KSS, and ROBoT-r Outcomes*

| *Variable* | *Alignment Reversal Score* | *Efficiency to Capture* | *Percentage of Successful Captures* |
| --- | --- | --- | --- |
| Mean RT | -.12 | .06 | .03 |
| Mean 1/RT | .02 | -.10 | -.05 |
| Lapses | -.21 | .02 | .00 |
| Slowest 10% RT | -.18 | -.01 | .03 |
| Fastest 10% RT | -.09 | .10 | -.03 |
| KSS | .03 | -.09 | .24* |

*Note*. **p* < .05. PVT = psychomotor vigilance test; RT = reaction time; KSS = Karolinska Sleepiness Scale

Table 2S. *Mixed-Effects Polynomial* *Regression Models for ROBoT-r Outcomes Controlling for Learning*

|  | *Trial Difficulty* | | | | | | | | | | | | | | | | | | |
| --- | --- | --- | --- | --- | --- | --- | --- | --- | --- | --- | --- | --- | --- | --- | --- | --- | --- | --- | --- |
|  | *Easy* | | | |  | *Medium-Easy* | | | |  | *Medium-Hard* | | | |  | *Hard* | | | |
| *Outcome* | *b*  (*SE*) | *p* | *R^2^_C_* | *R^2^_M_* |  | *b*  (*SE*) | *p* | *R^2^_C_* | *R^2^_M_* |  | *b*  (*SE*) | *p* | *R^2^_C_* | *R^2^_M_* |  | *b*  (*SE*) | *p* | *R^2^_C_* | *R^2^_M_* |
| Alignment-Reversal Score | -0.79 (0.82) | .33 | .18 | .01 |  | -2.03 (1.11) | .07 | .22 | .05 |  | -0.51 (1.39) | .71 | .17 | .01 |  | 0.12 (1.58) | .94 | .18 | .02 |
| Efficiency to Capture | -7.21 (14.44) | .62 | .39 | .01 |  | 36.53 (16.94) | .03* | .31 | .03 |  | 12.28 (18.75) | .51 | .24 | .01 |  | 30.67 (18.05) | .09 | .32 | .03 |
| Percentage of Successful Captures | 51.42 (42.52) | .23 | .96 | .95 |  | -4.40 (4.30) | .31 | .15 | .11 |  | 1.10 (3.37) | .74 | .09 | .05 |  | -1.21 (2.79) | .66 | .20 | .03 |
| Overall Success | -1.53 (2.70) | .57 | .06 | .03 |  | -2.25 (2.33) | .34 | .06 | .06 |  | .08 (2.11) | .97 | .04 | .01 |  | 0.55 (2.16) | .80 | .16 | .03 |

*Note*. Statistical results for quadratic function of time awake reported. SE = Standard Error of the Mean. *R^2^_C_* = Conditional Pseudo-*R^2^*. *R^2^_M_* = Marginal Pseudo-*R^2^*. **p* < .05.
